# Association of *APEX1* and *OGG1* gene polymorphisms with breast cancer risk among Han women in the Gansu Province of China

**DOI:** 10.1186/s12881-018-0578-9

**Published:** 2018-05-02

**Authors:** Tao Wang, Haitao Wang, Suisheng Yang, Hongyun Guo, Binming Zhang, Huan Guo, Lan Wang, Gongjian Zhu, Yongdong Zhang, Haihong Zhou, Xiuli Zhang, Haining Li, Haixiang Su

**Affiliations:** 1 Research Center of Translational Medicine, Gansu Provincial Academic Institute for Medical Research, NO. 2 Xiaoxihu East Street, Lanzhou, Gansu 730050 People’s Republic of China; 2 Department of Breast Surgery, Gansu Provincial Cancer Hospital, Lanzhou, Gansu 730050 People’s Republic of China

**Keywords:** *APEX1*, *OGG1*, Polymorphisms, Haplotype, Breast cancer

## Abstract

**Background:**

Genetic variations in key DNA repair genes may influence DNA repair capacity, DNA damage and breast carcinogenesis. The current study aimed to estimate the association of *APEX1* and *OGG1* polymorphisms with the risk of breast cancer development.

**Methods:**

A total of 518 patients with histopathologically confirmed breast cancer and 921 region- and age-matched cancer-free controls were genotyped for the *APEX1* polymorphisms rs3136817 and rs1130409 and the *OGG1* polymorphisms rs1052133 and rs2072668 using a QuantStudio™ 12 K Flex Real-Time PCR System.

**Results:**

The rs3136817 heterozygous TC genotype along with the rs3136817 dominant model (TC + CC) was strongly associated with breast cancer susceptibility (odds ratio [OR] = 0.670, 95% confidence interval [95% CI]: 0.513 - 0.873, *P* = 0.003; OR = 0.682, 95% CI: 0.526 - 0.883, *P* = 0.004, respectively). No significant associations were observed among rs1130409, rs1052133, rs2072668 and breast cancer risk. Furthermore, an allele combination analysis revealed that *APEX1* haplotypes containing C-T (alleles rs3136817 and rs1130409) conferred a significantly lower risk (corrected *P* < 0.001).

**Conclusion:**

This research is the latest report showing that an *APEX1* rs3136817 heterozygous genotype may have a positive influence on DNA repair capacity in patients with breast cancer and thus may have a potential protective effect for Chinese Han women.

**Electronic supplementary material:**

The online version of this article (10.1186/s12881-018-0578-9) contains supplementary material, which is available to authorized users.

## Background

A recent epidemiological report from the National Central Cancer Registry (NCCR) of China showed that breast cancer is the most commonly diagnosed cancer among women in China. For female breast cancer, the 5-year survival rate (73%) in China is considerably reduced compared with corresponding estimates in Australia (89%) and the United States (90%) and to a lesser degree in Europe (82%) [1]. Breast cancer ranks as the sixth leading cause of cancer deaths among women in rural areas of China, while the incidence rate (37.86/100,000) is continually increasing [2]. Numerous breast cancer susceptibility genes have been identified by molecular epidemiological studies of cancer (e.g., *BRCA1*/*2*, *TP53*, *PTEN* and *ATM*). *BRCA1* and *BRCA2* mutations comprise 32 to 82% of hereditary breast cancer cases [3]. The prevalence of these mutations, however, is considerably reduced in the Chinese population. For example, *BRCA1* and *BRCA2* mutations contribute to only 10% of hereditary breast cancer cases [4, 5]. Thus, identification of other novel susceptibility loci among Chinese breast cancer patients is needed. Analyses of genetic polymorphisms reveal correlations between breast cancer susceptibility and many loci [6, 7]. Recently, a considerable number of novel polymorphic variants have been identified by genome-wide association studies (GWAS) for breast cancer susceptibility [8, 9].

DNA repair systems act to safeguard the integrity of the genome against damage caused by exogenous and endogenous carcinogens and by mutagens. Defects in DNA repair capability can increase the risk of carcinogenesis [10], including breast cancer. Patients with breast cancer exhibit significantly reduced DNA repair proficiency [11]. The high accumulation of DNA damage may cause the initiation of carcinogenesis and aberrant cell division [12]. Additionally, several base excision repair (BER) enzymes may also participate in the regulation of other biological processes, including BER, cell cycle progression [13], transcription initiation [14] and apoptosis [15]. Moreover, 8-oxoguanine DNA glycosylase 1 (*OGG1*) and apurinic/apyrimidinic endonuclease 1 (*APEX1*) are key enzymes in the BER pathway of oxidative DNA damage, excising abasic residues. *OGG1* is a glycosidase that hydrolyzes the bonds between damaged bases and the sugar-phosphate backbone in DNA, creating an abasic site, while the endonuclease *APEX1* cleaves the 5′ end of the abasic site. Mutations in these genes are expected to lead to a mutation-prone phenotype and contribute to tumor formation [16]. In recent years, studies of breast cancer and DNA repair have emphasized the relationship between different single nucleotide polymorphisms (SNPs) in DNA repair-related genes and the probability of developing carcinoma. Kim et al. [17] reported that the combined effect of *APEX1* Asp148Glu was associated with an increased risk of breast cancer in a Korean population. Likewise, Roberts et al. [18] drew the conclusion that SNPs in nucleotide excision repair (NER) genes and BER genes affect the risk of developing breast cancer. Work by Smith et al. [19] demonstrated only a slight positive association between individual DNA repair genotypes and breast cancer risk; however, the combined effects of multiple polymorphisms in DNA repair pathways may be more noteworthy.

The present work aimed to investigate whether *APEX1* and *OGG1* gene polymorphisms (frequency ≥ 5%) exert any synergistic effects on breast cancer risk. Using HapMap data, we selected four putative functional tag SNPs in *APEX1* (rs1130409 and rs3136817) and *OGG1* (rs1052133 and rs2072668) and further evaluated the genetic interactions of these four polymorphisms and their relation to breast cancer risk among the study population of Han women living in the Gansu area in Northwest China.

## Methods

### Study participants and data collection

A total of 518 histopathologically confirmed patients with breast cancer were enrolled in this study from the Gansu Provincial Cancer Hospital (Gansu Province, China) between December 2014 and August 2017. None of these patients had been previously treated with systemic therapy. For the age- and region-matched controls, 921 cancer-free women were randomly selected from the Health Examination Surveys conducted in the same hospital. Inclusion criteria included negative history of all forms of cancer and no family history of breast cancer. All related data were drawn from questionnaires (see Additional file 1) and the patients’ medical charts. Informed consent was obtained from all individual participants, and this research was approved by the ethical committee of the Gansu Provincial Academic Institute for Medical Research and Gansu Provincial Cancer Hospital (IRB number: A201412040036).

### SNP identification and selection

Details of the SNP identification and selection have been described previously [20]. Briefly, tag SNPs can be used to identify most genetic variations existing in a gene and develop markers to assess the relationship between a disease and a specific region, regardless of whether the tag SNPs have functional effects [21]. Employing selection for tagger pairing processes, tag SNPs were selected from the HapMap CHB database (HapMap Data Rel 24/phase II Nov08). Tag SNPs were chosen by using Haploview version 4.0 software according to the following selection criteria: minor allele frequency (MAF) ≥ 0.05 and pair-wise r^2^ ≥ 0.8. Finally, the *APEX1* polymorphisms rs1130409 and rs3136817 and the *OGG1* polymorphisms rs1052133 and rs2072668 were selected.

### Genotyping method

A total of 5 mL of peripheral blood was collected in tubes containing EDTA from each of the participants for genomic DNA extraction using the genomic DNA Extraction Kit VER.3.0 according to the manufacturer’s instructions (TaKaRa Biotechnology, China). The DNA samples had A260/A230 ratios from 1.9 to 2.0 and A260/A280 ratios from 1.7 to 1.9; the concentrations of the samples were adjusted to 50 ng/μl. DNase/RNase-free distilled water was used in each assay as a non-template control (NTC). The multiplex TaqMan assays for genotyping were performed using the QuantStudio™ 12 K Flex Real-Time PCR System (Applied Biosystems, CA, USA) according to the manufacturer’s recommended operating conditions. The genotype data were analyzed using the OpenArray® SNP Genotyping Analysis software (version 1.0.5) (Applied Biosystems, CA, USA). To evaluate quality control, all genotyping assays were blind to the subject status. In addition, the reproducibility of genotyping was evaluated by direct sequencing in 20% of the samples randomly selected from each SNP and reached 100%.

### Statistical analysis

To assess the deviation of the genotype frequencies of the four tag SNPs from Hardy-Weinberg equilibrium (HWE) in the control subjects, the chi-square goodness-of-fit test was used. The distribution of the *APEX1* and *OGG1* genotypes were compared between controls and patients using Pearson’s chi-square test (goodness-of-fit) or Fisher’s exact test. For the association tests, multivariate unconditional logistic regression adjusting for age was performed to estimate odds ratios (ORs) and 95% confidence intervals (CIs) for the associations of the *APEX1* and *OGG1* polymorphisms with breast cancer risk and clinicopathologic features. As reported previously, classical genetic models, including dominant models and recessive models, were used to analyze the associations [22]. The frequency distributions of the *APEX1* and *OGG1* haplotypes were estimated using SHEsis online software (http://analysis.bio-x.cn/myanalysis.php) [23, 24].

To identify potential gene-gene interactions, we employed the multifactor dimensionality reduction (MDR) method (http://www.epistasis.org) to evaluate the best cross-group model for further confirmation. MDR is a robust and novel data-mining approach that was applied as described previously [25, 26]. Briefly, this method converts two or more discrete genetic variables to a single attribute. The aim of this approach is to identify the overall best combination all loci. With MDR, the newly formed one-dimensional variable can be assessed to predict disease status using cross-validation and permutation testing corrections by repeating the entire analysis on 1000 datasets. Furthermore, a final best MDR model is chosen that simultaneously has the maximal testing accuracy and cross-validation consistency (CVC).

To avoid false-positive results, haplotypes with frequencies greater than 3% were considered in all of the research participants. Bonferroni correction was employed to make the adjustment for multiple comparisons. All statistical analyses were performed using SPSS software (version 16.0), and differences were considered significant at *P* < 0.05.

## Results

### Demographic and pathological characteristics

All the detailed clinical and pathological characteristics of the study participants are presented in Table 1. The average age of the breast cancer patients was 49.3 (standard deviation [SD] = 8.2), and the average age of the controls was 48.4 years (SD = 8.9). No significant differences were observed between the two groups with respect to mean age and age distribution (*P* > 0.064). The distribution of all four SNPs in the control subjects obeyed the predicted Hardy-Weinberg equilibrium values (*P* > 0.05).

**Table 1:** Characteristics in breast cancer cases

Characteristics	Cases (%)
LN involvement	
Positive	179 (34.56)
Negative	237 (45.75)
Unknown	102 (19.69)
ER	
Positive	330 (63.71)
Negative	170 (32.82)
Unknown	18 (3.47)
PR	
Positive	266 (51.35)
Negative	230 (44.40)
Unknown	22 (4.25)
P53	
Positive	199 (38.42)
Negative	267 (51.54)
Unknown	52 (10.04)
Her-2	
Positive	117 (22.59)
Negative	379 (73.17)
Unknown	22 (4.25)
Ki-67	
Positive	298 (57.53)
Negative	172 (33.20)
Unknown	48 (9.27)
Age of onset	
≤ 35	46 (8.88)
> 35	472 (91.12)
Menopausal	
Premenopause	255 (49.23)
Postmenopause	163 (31.47)
Unknown	100 (19.31)

*LN* lymph node, *ER* estrogen receptor, *PR* progesterone receptor, *Her-2* human epidermal growth factor receptor 2, *Ki-67* monoclonal antibody Ki-67

### Association between genotypes and breast cancer risk

The genotypic and allelic distributions of all four SNPs are summarized in Table 2. The ancestral alleles were regarded as the reference group. In *APEX1* rs3136817, we found that the heterozygous TC genotype and the C allele were associated with breast cancer risk (adjusted OR = 0.670, 95% CI: 0.513 - 0.873, *P* = 0.003; adjusted OR = 0.729, 95% CI: 0.576 - 0.923, *P* = 0.009, respectively). Moreover, a decreased breast cancer risk was conferred by the combined rs3136817 genotypes (TC + CC) in the dominant model (adjusted OR = 0.682, 95% CI: 0.526 - 0.883, *P* = 0.004). However, no significant differences were found for overall genotype frequencies of rs1130409 in *APEX1* and the other two SNPs (rs2072668 and rs1052133) in *OGG1*.

**Table 2:** Genotype frequencies of *APEX1* and *OGG1* gene polymorphisms in controls and cases and their associations with breast cancer

SNPs	Genotype	Case *N* (%)	Control *N* (%)	OR (95% CI)^a^	*P* value
APEX1					
rs1130409	GG	91(17.57)	150(16.29)	Reference	
	GT	242(46.72)	417(45.28)	0.955(0.703-1.298)	0.770
	TT	185(35.71)	354(38.44)	0.864(0.629-1.187)	0.368
	G	424(40.93)	717(38.93)	Reference	
	T	612(59.07)	1125(61.07)	0.922(0.789-1.078)	0.309
	Dominant^b^			0.914(0.685-1.218)	0.538
	Recessive^c^			0.894(0.714-1.119)	0.328
rs3136817	TT	412(79.54)	668(72.53)	Reference	
	TC	99(19.11)	240(26.06)	0.670(0.513-0.873)	0.003
	CC	7(1.35)	13(1.41)	0.915(0.359-2.329)	0.851
	T	923(89.09)	1576(85.56)	Reference	
	C	113(10.91)	266(14.44)	0.729(0.576-0.923)	0.009
	Dominant			0.682(0.526-0.883)	0.004
	Recessive			1.002(0.394-2.547)	0.996
OGG1					
rs2072668	CC	83(16.02)	156(16.94)	Reference	
	CG	253(48.84)	440(47.77)	1.072(0.786-1.461)	0.661
	GG	182(35.14)	325(35.29)	1.056(0.763-1.459)	0.744
	C	419(40.44)	752(40.83)	Reference	
	G	617(59.56)	1090(59.17)	1.019(0.872-1.191)	0.811
	Dominant			1.065(0.795-1.427)	0.673
	Recessive			1.002(0.799-1.258)	0.984
rs1052133	CC	87(16.80)	154(16.72)	Reference	
	CG	243(46.91)	431(46.80)	0.986(0.725-1.342))	0.928
	GG	188(36.29)	336(36.48)	0.985(0.715-1.356)	0.925
	C	417(40.25)	739(40.12)	Reference	
	G	619(59.75)	1103(59.88)	0.993(0.850-1.161)	0.934
	Dominant			0.985(0.738-1.317)	0.921
	Recessive			0.995(0.795-1.246)	0.966

*OR* odds ratio, *CI* confidence interval;

^a^ORs were adjusted for age;

^b^The dominant model: comparing the combination of heterozygotes and minor allele homozygotes with the major allele homozygotes;

^c^The recessive model: comparing minor allele homozygotes with the combination of heterozygotes and major allele homozygotes

### Association between genotypes and clinicopathological features

We investigated the association between the genotypes of each SNP and the clinicopathological features of the breast cancer patients, including progesterone receptor (PR), estrogen receptor (ER), P53 protein, Ki67 protein, human epidermal growth factor receptor 2 (Her-2), staging and lymph node metastasis. Only statistically significant results are presented for rs1130409 and rs2072668 in Table 3. In order to facilitate to compare data of subtype study, related data of rs3136817 which associated with the risk of breast cancer were also added in Table 3. For the *APEX1* polymorphism rs1130409, the frequency of the GT and TT genotypes was increased in ER-positive patients compared with the GG genotype (adjusted OR = 1.709, 95% CI: 1.028 - 2.842, *P* = 0.039; adjusted OR = 1.725, 95% CI: 1.016 - 2.930, *P* = 0.043, respectively), and rs1130409 was also associated with ER-positive patients in the dominant model (adjusted OR = 1.716, 95% CI: 1.068-2.759, *P* = 0.026). Furthermore, compared with the GG genotype, the TT homozygous genotype frequency was decreased in Her-2-positive patients (adjusted OR = 0.508, 95% CI: 0.281 - 0.917, *P* = 0.025). For the *OGG1* polymorphism rs2072668, compared with the CC genotype, the homozygous GG genotype showed a reduced frequency in Ki-67-positive patients (adjusted OR = 0.536, 95% CI: 0.297 - 0.968, *P* = 0.039).

**Table 3:** Clinicopathological features and *APEX1* and *OGG1* gene polymorphisms

Clinical features	SNPs	Genotype	*N* (%)		OR (95% CI)^a^	*P* value
			Positive	Negative		
APEX1						
ER	rs1130409	GG	48(14.55)	38(22.35)	Reference	
		GT	158(47.88)	74(43.53)	1.709(1.028-2.842)	0.039
		TT	124(37.58)	58(34.12)	1.725(1.016-2.930)	0.043
		Dominant^b^			1.716(1.068-2.759)	0.026
		Recessive^c^			1.174(0.796-1.732)	0.417
	rs3136817	TT	267(80.91)	130(76.47)	Reference	
		TC	59(17.88)	37(21.76)	0.779(0.491-1.236)	0.288
		CC	4(1.21)	3(1.76)	0.673(0.148-3.063)	0.608
		Dominant			0.771(0.492-1.207)	0.255
		Recessive			0.708(0.156-3.213)	0.655
Her-2	rs1130409	GG	27(23.08)	59(15.57)	Reference	
		GT	56(47.86)	176(46.44)	0.689(0.399-1.190)	0.182
		TT	34(29.06)	144(37.99)	0.508(0.281-0.917)	0.025
		Dominant			0.608(0.364-1.015)	0.057
		Recessive			0.663(0.423-1.040)	0.074
	rs3136817	TT	95(81.20)	298(78.63)	Reference	
		TC	21(17.95)	75(19.79)	0.875(0.512-1.497)	0.627
		CC	1(0.85)	6(1.58)	0.505(0.060-4.265)	0.531
		Dominant			0.848(0.501-1.433)	0.537
		Recessive			0.519(0.062-4.365)	0.546
Ki-67	rs3136817	TT	239(80.20)	134(77.91)	Reference	
		TC	54(18.12)	36(20.93)	0.842(0.525-1.350)	0.475
		CC	5(1.68)	2(1.16)	1.451(0.277-7.610)	0.660
		Dominant			0.873(0.552-1.383)	0.564
		Recessive			1.502(0.287-7.854)	0.630
OGG1						
Ki-67	rs2072668	CC	55(18.46)	21(12.21)	Reference	
		CG	146(48.99)	82(47.67)	0.675(0.381-1.195)	0.178
		GG	97(32.55)	69(40.12)	0.536(0.297-0.968)	0.039
		Dominant			0.612(0.355-1.052)	0.076
		Recessive			0.913(0.620-1.344)	0.644

*ER* estrogen receptor, *Her-2* human epidermal growth factor receptor 2, *Ki-67* monoclonal antibody Ki-67, *OR* odds ratio, *CI* confidence interval;

^a^ORs were adjusted for age;

^b^The dominant model: comparing the combination of heterozygotes and minor allele homozygotes with the major allele homozygotes;

^c^The recessive model: comparing minor allele homozygotes with the combination of heterozygotes and major allele homozygotes

### Haplotype analyses

We further investigated haplotype frequencies in the patients and controls. Only haplotypes with a frequency greater than 5% are presented in Table 4. In this combination, haplotype CT in *APEX1* had a lower frequency in patients compared with controls. After applying the Bonferroni multiple adjustment, only one significant *P* value was observed at the *APEX1* CT haplotype (*P* < 0.001), indicating a significant difference. In addition, no statistically significant differences between controls and patients were observed in the frequencies of other *APEX1* and *OGG1* haplotypes.

**Table 4:** Frequency distributions of haplotypes of *APEX1* and *OGG1* in cases and controls

Haplotype	Case (freq%)	Control (freq%)	*P* value^c^	OR (95% CI)	*P*c
APEX1^a^					
CG	94.19 (9.10%)	179.03 (9.70%)	0.581	0.929 (0.715-1.207)	NS
CT	18.81 (1.80%)	86.97 (4.70%)	7.10e-05	0.373 (0.225-0.618)	< 0.001
TG	329.81 (31.80%)	537.97 (29.2%)	0.140	1.132 (0.960-1.335)	NS
GG	593.19 (57.30%)	1038.03 (56.40%)	0.639	1.038 (0.890-1.210)	NS
OGG1^b^					
CC	403.81 (39.00%)	728.87 (39.60%)	0.908	0.991 (0.847-1.159)	NS
CG	15.19 (1.50%)	23.13 (1.30%)	–	–	NS
GC	13.19 (1.30%)	10.13 (0.50%)	–	–	NS
GG	603.81 (58.3%)	1079.87 (58.6%)	0.908	1.009 (0.862-1.181)	NS

*Pc* corrected *P* value (after Bonferroni multiple adjustment), *OR* odds ratio, *CI* confidence interval, *NS* not significant

^a^The order of SNPs in *APEX1* is rs3136817 and rs1130409

^b^The order of SNPs in *OGG1* is rs2072668 and rs1052133

^c^*P* value calculated by Fisher’s exact test

### Interaction analysis

We further adopted the analytical method of MDR data mining to examine the potential interactions among the four polymorphisms within the *APEX1* and *OGG1* genes. Table 5 shows the best interaction models identified by the MDR analysis, with testing balance accuracy (TBA) and cross-validation consistency. *APEX1* rs3136817, as the best one-locus model, had the maximum testing-balanced accuracy (0.5323) and maximum consistency (100%) among the four SNPs. A four-locus model (rs2072668, rs1052133, rs3136817 and rs1130409) also exhibited maximum consistency (100%), but its TBA (0.5029) was lower than that of the one-locus model. Moreover, the combination of rs3136817 and rs2072668 formed the best two-locus model, with a high TBA (0.5291) and CVC (70%), among all combinations of two SNPs. The combination of rs3136817, rs2072668 and rs1130409 produced the best three-locus model, with a high TBA (0.5044) and CVC (90%), among all pair-wise combination of three SNPs. However, no significant association was observed for these interaction models out of 1000 permutations.

**Table 5:** MDR interaction analysis between SNP-SNP

Each overall best model	Testing Balance Accuracy	CVC^a^	*P* Value^b^
One-locus: rs3136817	0.5323	10/10	0.1419
Two-locus: One-locus plus rs2072668	0.5291	7/10	0.3846
Three-locus: Two-locus plus rs1130409	0.5044	9/10	0.7383
Four-locus: Three-locus plus rs1052133	0.5029	10/10	0.7502

^a^CVC cross-validation consistency

^b^*P*-values as calculated after 1000 permutations

## Discussion

Genetic polymorphisms in key genes involved in DNA repair may influence DNA damage response, carcinogenesis and DNA repair capacity. Polymorphic variants have been confirmed to be good candidates for assessing cancer risk. We estimated the relationship between *APEX1* and *OGG1* gene polymorphisms and breast cancer risk among 1430 Han women of Northwest China in this experiment using a tag SNP-based study. The key finding was that the *APEX1* polymorphism rs3136817 might mediate synergistic and independent effects on breast carcinogenesis. We found that the rs3136817 heterozygous TC genotype and the combined genotype (TC + CC) were associated with decreased breast cancer risk.

DNA excision repair capacity is known to play a crucial role in carcinogenesis [27]. If changes that occur in the DNA sequence due to copying errors are not corrected, they may ultimately interfere with cell function. Damaged or inappropriate bases can be repaired by several mechanisms. The BER pathway is considered the primary mechanism involved in protecting against gene mutations and repairing DNA damage, and *APEX1* and *OGG1* are key components of this pathway. The BER proteins encoded by these genes act in a highly coordinated manner at the site of DNA damage. For example, repair of 8-oxoguanine, an oxidized base, is initiated by the *OGG1* glycosylase, which recognizes and removes damaged bases, forming an apurinic site that is cleaved by *APEX1.* The resulting single-strand break can be subsequently repolymerized by DNA ligase 3 and polymerase *β* [28]. According to our current results, patients carrying the rs3136817 TC genotype had a reduced breast cancer risk, implying that heterozygosity at rs3136817 may have a positive influence on DNA repair capacity in DNA damage responsive pathways and thus potentially prevent breast cancer. Similar results were observed in bladder cancer and lung cancer. Nevertheless, this protection was not observed in individuals carrying other SNPs. Similar researches were only reported in bladder cancer and lung cancer. Zhu et al. [29] reported that APEX1 rs3136817 TC genotype was associated with a decreased risk of bladder cancer. However, Li et al. [30] demonstrated that no association between APEX1 rs3136817 and the risk of radiation-induced pneumonitis grade ≥ 3. Our study is the first to demonstrate that the rs3136817 heterozygous genotype was associated with a decreased risk of breast cancer. Of all the SNPs assessed, the rs1130409 and rs1052133 polymorphisms showed the most consistent relationship to breast cancer in two previous reports, demonstrating that these variations have no significant impact on breast cancer risk [18, 31]. However, a few reports which were different from our results also indicated that breast cancer risk was significantly associated with APEX1 rs1130409 in North Indian [32], Korean [17] and Caucasian [19] women. To the best of our knowledge, the rs3136817 polymorphic locus has not been previously evaluated regarding its association with cancer, except for bladder cancer and lung cancer. Moreover, the possibility that the associations noted above might have occurred by chance cannot be excluded. Further evidence in different regional populations and larger sample sizes, in addition to functional studies, are required to reinforce these results.

We further found that the *APEX1* rs1130409 GT and TT genotypes were increased in ER-positive patients by analyzing the association of clinicopathological features with the four SNPs, suggesting that these genotype carriers exhibited adverse clinicopathological features of breast cancer and failed to benefit from endocrine therapy. In addition, a lower frequency of women carrying the TT genotype was Her-2 positive. Her-2 is a ligand-less member of the human epidermal growth factor receptor family. Approximately 15% of patients with breast cancer exhibit Her-2 over-expression, which is associated with invasive behavior, unresponsiveness to common endocrine therapies, poor prognosis and reduced survival [33, 34]. For *OGG1*, the rs2072668 polymorphism was also associated with the pathologic characteristics of patients. The rs2072668 homozygous GG genotype carriers had a reduced frequency of Ki-67-positive expression. The Ki-67 protein is a nuclear marker of cell proliferation that is expressed at high levels in breast cancer patients, and increased expression is associated with worse outcomes [35, 36]. Recent studies have indicated that long-term outcomes may be predicted by changes in Ki-67 expression after endocrine treatment [37]. Therefore, our current results indicate that the *APEX1* rs1130409 TT genotype and the *OGG1* rs2072668 GG genotype deficiencies may lead to poorer prognosis and reduced survival.

In our study, the associations between breast cancer risk and haplotypes were also assessed. The *APEX1* haplotype containing C-T (alleles rs3136817 and rs1130409) was observed at a higher frequency in the controls. Consequently, we infer that the *APEX1* CT haplotype may be involved in decreasing the risk of breast cancer. MDR analysis has been used to examine the interactions of multiple genes in common diseases as a promising data-mining approach because it easily overcomes some of the limitations and inadequacies of traditional statistics, such as logistic regression, to characterize and examine gene-gene and gene-environment interactions. Meanwhile, a four-locus interaction associated with cumulative breast cancer risk was identified by MDR analysis. However, the four-locus best interaction model did not exhibit significant improvements compared with the other models in this case. The main effect model of rs3136817 performed best, which implies that the *APEX1* rs3136817 polymorphism is a powerful risk factor and may play an important role in the interaction with other SNPs in affecting breast cancer development, either synergistically or antagonistically. Nevertheless, no statistical significance was observed, and evidence is therefore needed to support the hypothesis of SNP-SNP interactions in the future.

## Conclusions

In summary, our data provide clear evidence that the rs3136817 polymorphism in *APEX1* and a corresponding haplotype may be involved in breast cancer risk in the Han women of Northwest China. Notably, the rs3136817 heterozygous variant exhibited enhancement in the major DNA repair pathway, and this capacity may prevent the early development of breast cancer. As far as we know, this study is the latest to report that the *APEX1* rs3136817 genotype is associated with cancer risk. The findings further suggest that the combined effect of SNPs determines the individual women’s risk for breast cancer. A better understanding of the mechanism of carcinogenesis will facilitate improved, individualized pharmaceutical therapy for patients with breast cancer and the implementation of breast cancer prevention strategies.

## Additional file

Additional file 1: A questionnaire survey of breast health. Questionnaire included participant's eating habits, living environment, lifestyle, smoking, physiological state, reproductive condition, past medical history and family history of cancer. (PDF 415 kb)

## Abbreviations

APEX1: Apurinic/apyrimidinic endonuclease 1
BER: Base excision repair
CIs: Confidence intervals
CVC: Cross-validation consistency
ER: Estrogen receptor
GWAS: Genome-wide association studies
Her-2: Human epidermal growth factor receptor 2
HWE: Hardy-Weinberg equilibrium
MDR: Multifactor dimensionality reduction
NCCR: National Central Cancer Registry
NER: Nucleotide excision repair
NTC: Non-template control
OGG1: 8-oxoguanine DNA glycosylase 1
ORs: Odds ratios
PR: Progesterone receptor
SD: Standard deviation
SNPs: Single nucleotide polymorphisms
TBA: Testing balance accuracy
