# Supplementary material for: Association of APEX1 and OGG1 gene polymorphisms with breast cancer risk among Han women in the Gansu Province of China
Source: BMC Med Genet. 2018 May 2;19:67. doi: 10.1186/s12881-018-0578-9 (PMC5930440; doi:10.1186/s12881-018-0578-9)
Supplement: Supplementary file 1 — A questionnaire survey of breast health. Questionnaire included participant's eating habits, living environment, lifestyle, smoking, physiological state, reproductive condition, past medical history and family history of cancer. (PDF 415 kb) [file 12881_2018_578_MOESM1_ESM.pdf]

受访编号：09　01 |__|__| |__|__| |__|__|__|__|
省　市　区　街道　序 号

DNA 损伤修复相关基因多态性与乳腺癌易感性的研究项目

风险评估问卷

姓名：____________

性别：______ 1.男 2.女

出生日期：___________年______月______日（请填写阳历生日）

民族：______ 1.汉族　2.蒙古族　3.回族　4.满族　5.壮族

6.维吾尔族　7.哈萨克族　8.其他，请注明__________

籍贯：________ 省 ________ 市 ________ 县（区）

身份证号：|__|__|__|__|__|__|__|__|__|__|__|__|__|__|__|__|__|__|

联系电话：|__|__|__|__|－|__|__|__|__|__|__|__|（固定电话）

|__|__|__|__|__|__|__|__|__|__|__|（手机）

常住地址：______________________________________

工作单位：______________________________________

DNA 损伤修复相关基因多态性与乳腺癌易感性的研究项目

知情同意书

本项目是一个试验性研究工作，其研究目的是拟用 TaqMan OpenArray 基因分型系统联合检测与乳腺癌相关的多个 DNA 损伤修复相关基因及其 SNPs。通过大样本研究,探讨 DNA 修复过程中存在的单基因 SNPs 的协同作用，多基因 SNPs 的交互作用机制，阐明 DNA 损伤基因多态性与乳腺癌易感性的相关性，阐明多基因 SNPs 间的相互作用。

甘肃省肿瘤发病调查显示乳腺癌居甘肃妇女肿瘤发病首位。机体内的 DNA 损伤修复系统对预防癌症的发生起到关键作用，当 DNA 受到损伤后，机体可启动该系统进行修复，以维持遗传物质的正常功能。DNA 修复能力的个体差异是决定肿瘤易感性的重要因素，修复基因单核苷酸多态性(SNPs)又是导致 DNA 修复能力个体差异的重要原因。本项目的研究为乳腺癌预防、诊断及个体化治疗提供科学依据；有助于降低乳腺癌的发病及死亡率，提高患者生存率。

参加本项目后，首先要填写《风险评估问卷》，然后我们将邀请您参加乳腺癌的相应筛查。筛查的主要方法如下:

乳腺癌：40-44 岁者，乳腺超声检查（有可疑发现者加 X 线检查）。45 岁以上者，乳腺超声+X 线

参加本项目的好处:

我们将免费对您进行风险评估，将免费为您进行相关检查。通过检查，您有可能被发现患有某些疾病，包括癌症或癌前病变，这样就可以得到早期诊断和治疗的机会。由于经费有限，发现不正常的情况后，进一步检查和治疗的费用不由本项目负担。

参加本项目的风险:

1. 任何临床检查手段，尤其是影像学检查，都无法做到百分之百准确。如果影像学检查显示有异常情况发生，需要进一步的确诊。
2. 乳腺 X 线检查对身体有一定的辐射，但剂量较低，对一般人群是安全的。
3. 癌症的发病原因是复杂的，包括饮食、环境、免疫、内分泌、遗传等因素。目前对于癌症的研究尚不能确定每个人患癌的真正病因，对某一个体患某种癌的风险评估是基于流行病学为基础的以常见危险因素作为评判标准进行的。随着研究的深入，可能会发现新的危险因素。

参加本项目的义务:

您需要提供真实的问卷信息，按照我们的安排进行各项检查，配合我们进行以后开展的随访及健康管理工作。

保密原则:

您的所有信息（流行病学信息、检查结果等）我们都会为您保密。信息由专人负责保管和录入。保证不会泄露给第三方。

自愿原则:

您参加本项目完全是自愿的。在项目的任何阶段，您都有权利随时退出项目。

自我声明:

我已阅读本知情同意书，理解全部相关情况，一些问题已经同项目组工作人员进行了讨论并得到满意答复，我同意参加这项研究。

参加人签字:　　　　　　　　　　　　日期:　　　年　　月　　日

工作人员声明:

我已向调查对象宣传和解释了这份知情同意书，他/她已经理解并同意参加本项目。

工作人员签字:　　　　　　　　　　　日期:　　　年　　月　　日

A. 基础信息

A01. 身高: ______ 厘米　A02. 体重: ______ 公斤　A03. 腰围: ______ 厘米

A04. 文化程度: 1.未受教育　2.小学　3.初中　4.高中/中专/技校　5.大专　6.大学及以上 □

A05. 婚姻状况: 1.未婚　2.初婚　3.再婚　4.离异　5.丧偶　6.未婚同居 □

A5.1 如有婚史，您初次结婚的年龄（周岁）: □□

A06. 您的职业: 1.专业技术人员 2.国家机关和企事业单位责任人 3.职员 4.商人 5.农民 6.工人 7.服务性工作人员 8.家务 9.其他，请注明_______ □

A07. 您是否有有害物质职业接触？　0.否（跳转至 B01）　1.是 □

A7.1 职业接触何种有害物质（可多选）？1.石棉 2.橡胶 3.煤尘、粉尘 4.农药 5.发射线 6.铍、铀、氡等 7.其他，请注明 __________ □□□□□□

B. 饮食习惯

B01. 近两年来，您个人下述食物的平均摄入量（可从家庭总量除以人口数计算出个人平均量）？

B1.1 新鲜蔬菜（不包括土豆，未烹饪的生重）: 0.从不吃 1. <5 斤/周 2. ≥5 斤/周 □

B1.2 新鲜水果（未去皮的生重）: 0.从不吃 1. <2.5 斤/周 2. ≥2.5 斤/周 □

B1.3 畜肉(指猪、牛、羊肉，未烹饪的生重): 0.从不吃 1. ≤7 两/周 2. >7 两/周 □

B1.4 粗粮(除白面、大米，未烹饪的生重): 0.从不吃 1. <1 斤/周 2. ≥1 斤/周 □

B02. 您平时的饮食喜好习惯:

B2.1 口味: 1.重盐　2.适中　3.清淡 □

B2.2 油质: 1.油脂较高　2.适中　3.油脂较低 □

C. 生活环境、方式和习惯

C01. 您近十年来的生活环境是否存在较严重空气污染（如果长期在大城市生活则直接被认定有空气污染）？　0.否　1.是 □

C02. 您是否吸烟（每天吸一支以上并连续或累计 6 个月以上者定义为吸烟）？
0.否，从不吸（跳转至 C03）　1.是，目前仍在吸　2.以前吸，目前已戒烟 □

C2.1 如果您仍在吸烟或曾吸烟，平均每天吸烟多少支（1 两烟叶≈50 支卷烟）？ □□

C2.2 如果您仍在吸烟或曾吸烟，扣除戒烟年数，共吸烟多少年（不足一年按一年计）？

C2.3 如果您目前已戒烟，这次戒烟已持续多少年（不足一年按一年计）？

C03. 您长期居住和\或工作的室内环境中，是否有人经常吸烟？0.否（转至C04）1.是

C3.1 如果是，那么在有烟雾室内环境中，您居住和\或工作了多少年（不足一年按一年计）？

C04. 您是否经常参加体育锻炼（经常是指平均每周3次以上，每次超过30分钟）？

0.否　1.是

D. 心理和情绪

D01. 近些年您是否曾经受较大的精神创伤（亲人患重病或死亡、家庭不和破裂、重大财产损失、意外失业、重大意外身体伤害、暴力恐吓等）？ 0.否　1.是

D02. 近些年您是否有较长一段时间内精神处于压抑状况（连续超过6个月)？

0.否　1.是

E. 生理和生育

E01. 您的首次月经年龄是?

E02. 您的月经周期是否规律?　0.否　1.是

E03. 您是否已经闭绝经?　0.否（跳转至E04）　1.是

E3.1 如果是，那么您的停经年龄是（周岁):

E04. 您是否分娩（包括自然分娩和人工分娩）?　0.否（跳转至E05）　1.是

E4.1 如果是，那么您的初次生育年龄是（周岁):

E05. 您是否患有良性乳腺疾病（乳腺增生、结节、导管扩张、良性纤维腺瘤、感染等）?

0.否　1.是，请注明疾病名称: ______________

E06. 您是否曾进行女性生殖系统手术（子宫、卵巢和输卵管等部位的手术，包括剖腹产）?

0.否　1.是，请注明手术名称或手术部位: ______________

E07. 您是否有血缘亲属曾患乳腺癌?　0.否　1.是

E08. 您是否有血缘亲属曾患卵巢癌?　0.否　1.是

F. 既往史

F01. 您是否曾被确诊患有任何癌症（非黑色素瘤性皮肤癌除外）？

0.否（跳转至 F02） 1.是 ☐

F1.1 如果是，那么是何种癌症________________

F02. 您是否有其他系统性疾病？

F1.1 高血压： 0.否 1.是 ☐ F1.2 高血脂症：0.否 1.是 ☐

F1.3 糖尿病： 0.否 1.是 ☐ F1.4 其他：0.否 1.是，请注明____ ☐

G. 恶性肿瘤家族史

G01. 您的血缘亲属中，是否有人曾患癌症？ 0.否 1.是 ☐

如果您确认 A-G 项的所有问题填选真实准确，请**签名**：____________

☐☐☐☐ 年 ☐☐ 月 ☐☐ 日

调查员编号：______ 签名：__________ ；审核员编号：______ 签名：__________

风险评估问卷填写说明

封面、受访编号和个人信息

个人信息请如实填写,其中出生年月日为阳历,联系电话可以在手机和固话中二选。

A、基础信息

A01-A03、身高、体重、腰围填写单位为“厘米/cm”、“公斤/kg”、厘米/cm”,数值以受访者最近测量值为准。除“体重”可保留到小数点后 1 位外,“身高”和“腰围”需为整数。

A04、本题为单选题,文化程度以被评估人最高学历为准。

A05、本题为单选题,依据被评估人目前的婚姻状况进行填写。

A5.1、如有婚史,请填写初次结婚时的周岁年龄。

A06、本题为单选题,职业填选为被评估人目前从事职业或退休前从事的职业。

A07、本题为单选题,这里的有害物质职业接触是指劳动者在职业活动过程中长期反复接触的有害物质。

A7.1、本题为多选题,选项可同时选择。

B、饮食习惯

B01、本题为单选题,长期以来个人食物平均摄入量,可从家庭总量除以人口数计算个人平均量,所有食物重量均已其未烹任(新鲜水果为未去皮)时的重量计算。

B02、本题为单选题,依照目前被评估人平时的饮食喜好习惯酌情填写。

C、生活环境、方式和习惯

C01、本题为单选题,长期生活环境空气污染主要指长期生活在大城市或生活居住场所 500 米范围内存在空气污染源,如化工厂。

C02、本题为单选题,每天吸一支以上并连续或累计 6 个月以上者定义为吸烟。

C2.1-C2.3、请如实填写准确的整数数字。

C03、本题为单选题,“经常”在这里是指平均每周至少 1 次,连续 6 个月以上。

C3.1、请如实填写准确的整数数字。

C04、本题为单选题,“体育锻炼”在这里主要指慢跑、游泳、骑自行车、太极拳、篮球、竞走等有氧运动,散步有益于身心健康,单在这里不属于体育锻炼。平均每周 3 次以上,每次超过 30 分钟视为“经常”。

D、心理和情绪

D01、本题为单选题,“精神创伤”包括人患重病或死亡、家庭不和破裂、重大财产损失、意外失业、重大意外身体伤害、暴力恐
吓等可能引起人体强烈应激反应的生活事件

D02、本题为单选题,“较长一段时间”一般指超过 6 个月

E、女性生理和生育

E01、依据被评估人实际情况填写,年龄为周岁。

E02、本题为单选题。

E03、本题为单选题,依据被评估人目前状况填写。

E04-E05、本题为单选题,年龄为周岁。

E06、这里的“女性生殖系统手术”主要指子宫、卵巢和输卵管等内生殖器官部位的手术,包括剖腹产,不包括人工流产。

E07-E08、乳腺癌及卵巢癌家族史,根据实际情况填选。

F、既往史

这里的“既往史”是指经由具备相应检查条件和诊断能力的正规医疗机构检查并由执业医师确诊的疾病,不能由被评估人根据主观臆断填写。

E01、癌症病史须由具备相应资质的医疗机构和专业人员确诊,不包括非黑色素瘤性皮肤癌

G、恶性肿瘤家族史

根据实际情况填选。
